# The ecological threat posed by invasive species as silent carriers of global priority bacteria to wildlife

**DOI:** 10.1016/j.onehlt.2025.101043

**Published:** 2025-04-18

**Authors:** Gabriel Siqueira dos Santos, Fábio Parra Sellera, João Pedro Rueda Furlan, José Soares Ferreira Neto, Marcos Bryan Heinemann

**Affiliations:** a Departamento de Medicina Veterinária Preventiva e Saúde Animal, Faculdade de Medicina Veterinária e Zootecnia, Universidade de São Paulo, São Paulo, Brazil; b Departamento de Clínica Médica, Faculdade de Medicina Veterinária e Zootecnia, Universidade de São Paulo, São Paulo, Brazil; c Faculdade de Medicina Veterinária, Universidade Metropolitana de Santos, Santos, Brazil; d Departamento de Microbiologia, Imunologia e Parasitologia, Escola Paulista de Medicina, Universidade Federal de São Paulo, São Paulo, Brazil

## Abstract

• Invasive species can act as silent carriers of multidrug-resistant bacterial species.
• Invasive species in natural environments without predators can amplify the spread of antimicrobial resistance.
• Global data on WHO priority bacteria and antimicrobial resistance in invasive species are provided.
• Epidemiological surveillance of antimicrobial resistance in invasive species is discussed.

The introduction of non-native animal species to new biomes has caused numerous ecological health burdens across the globe. Indeed, the Thematic Assessment Report from the Intergovernmental Science-Policy Platform on Biodiversity and Ecosystem Services (IPBES) documents 37,000 invasive species (IS) worldwide, with approximately 3500 causing significant impacts and 200 new ones recorded annually [1]. In addition to agricultural damage and threats to native biodiversity due to competition for resources and territories, IS can also be responsible for cross-transmit microbial pathogens [2].

Accordingly, the IPBES and scientific literature have highlighted the role of IS in transmitting zoonotic pathogens, including *Mycobacterium* spp., *Leptospira* spp., and Rabies virus [1,3,4]. The ability of IS to adapt to various environmental conditions and exhibit exponential growth, sometimes outcompeting native wild species, highlights their potential to introduce new pathogens into several ecosystems or amplify those already established [3]. On the other hand, the emergence of antimicrobial resistance (AMR), especially of World Health Organization (WHO) priority bacterial pathogens [5], mediated by IS have received less attention. Therefore, we systematically evaluated the global scientific literature documenting the occurrence of WHO-priority bacteria in IS.

We found limited epidemiological, microbiological, and genomic data on this issue. Particularly concerning is the lack of research on the occurrence of WHO high- and critical-priority bacteria in these species. Remarkably, an increasing number of reports have identified methicillin-resistant *Staphylococcus aureus* (MRSA) and extended-spectrum β-lactamase (ESBL) and/or carbapenemase-producing *Enterobacterales* in native wildlife populations. These important healthcare-associated pathogens [6] remain under-explored in the context of IS (Fig. 1; Supplementary Table S1).

**Fig. 1:**
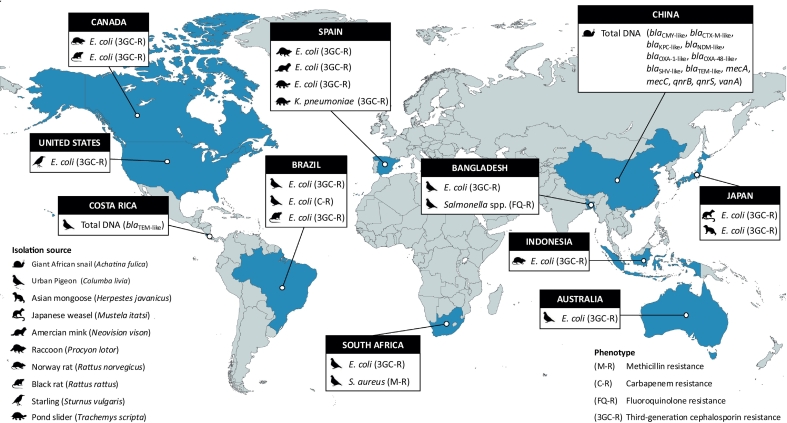
Global distribution of WHO bacterial priority pathogens and related genes reported in invasive species. Data were retrieved from PubMed database via the National Center for Biotechnology Information (NCBI) interface. Third-generation cephalosporin resistance, 3GC-R; Carbapenem resistance, C-R; Fluoroquinolone resistance, FQ-R; Methicillin resistance, M-R.

We identified that most studies investigated the presence of such pathogens in invasive synanthropic animals (e.g.*,* pigeons and rodents). In Brazil, MRSA and international clones of *Escherichia coli* (i.e.*,* ST10, ST224, and ST457) producing ESBLs were isolated from pigeons (*Columba livia*) and black rats (*Rattus rattus*) (Supplementary Table S1). Originally from Europe, North Africa, and parts of Asia, these animals have spread to various parts of the world [7]. Although these invasive synanthropic species are already integrated into the local fauna in many countries, their coexistence in urban and rural areas and their consumption of human food waste can serve as pathways for the acquisition of bacteria and subsequent spillover into several environments [8].

The migratory European starling (*Sturnus vulgaris*), a harmful invasive species in the United States, has been associated with the dissemination of clones of multidrug-resistant (MDR) and ESBL-producing *E. coli* to farm animals in animal feeding operations [9]. Indeed, flying birds have the potential to rapidly spread clinically relevant bacteria and AMR determinants over long distances, while the issue of AMR in invertebrates has been even more neglected [10]. Furthermore, the MecA and OXA-72 determinants that confer resistance to methicillin and carbapenems, both last-resort antimicrobials, were identified in giant African snails (*Lissachatina fulica*); (Supplementary Table S1). Accordingly, the urbanization could influence the gut microbiome of these invasive snails, suggesting that they carry higher levels of biological and xenogenetic pollutants compared to native snails [11].

Another controversial issue is the role of native wild species, recognized for their potential to carry AMR bacteria and possibly become important IS in other countries. For instance, wild boars (*Sus scrofa*) and European rabbits (*Oryctolagus cuniculus*), which move between urban, rural, and wild areas, have been identified as wild carriers of medically significant antimicrobial-resistant bacteria in countries where they are native [12,13]. In other countries, such as Brazil [14] and Australia [15], these species are potentially harmful IS. However, their role as carriers of AMR-related bacteria has not yet been investigated in the context of IS.

The importation of exotic pets and their subsequent release or escape into the wild, as exemplified by Burmese pythons (*Python bivittatus*) in Florida, United States, is another significant concern [16]. A case study in Germany found mobile colistin resistance gene *mcr-1* and ESBL-encoding genes in *E. coli* strains taken from the gut microbiota of Asian grass lizards (*Takydromus sexlineatus*) [17]. It is worth noting that there are few studies that look at the international trade of exotic animals as a possible way for AMR to spread to new areas and clinical and ecological impacts of the introduction of human-associated MDR bacterial pathogens into native wildlife populations.

Anthropogenic actions have significantly contributed to the global spread of IS, facilitating their introduction into new environments [18]. Our understanding of wildlife microbiota is notably limited, and the scarcity of studies in this field suggests that many pathogens and even AMR determinants have yet to be discovered. The lack of knowledge increases the potential for spreading these microorganisms across various hosts. Moreover, human activities, such as international trade, habitat destruction, and climate change, have created pathways for these non-native species to establish themselves in wildlife populations worldwide. For instance, the global movement of goods and live animals can unintentionally transport IS to new locations, allowing them to thrive and outcompete native species.

Additionally, habitat alterations, such as deforestation and urbanization, often disrupt local ecosystems and make them more susceptible to invasion. Climate change further exacerbates the issue by altering habitats and making previously unsuitable areas more hospitable to IS [19]. These combined factors have led to the widespread dissemination of IS, posing significant threats to biodiversity and ecosystem stability across the globe. Thus, considering that we still do not fully understand which bacteria and even AMR determinants IS may be carrying and the potential for these agents to spread among native fauna, this gap in knowledge merits closer scrutiny.

The complexity of these issues necessitates a thorough debate by ecological authorities and the scientific community, as they have unpredictable ecological consequences. Given that AMR and IS have separately threatened global health, an integrative approach under the One Health initiative should monitor their combined impacts to better understand their potential for causing ecological imbalances.

## CRediT authorship contribution statement

**Gabriel Siqueira dos Santos:** Writing – review & editing, Writing – original draft, Visualization, Formal analysis, Conceptualization. **Fábio Parra Sellera:** Writing – review & editing, Writing – original draft, Formal analysis, Conceptualization. **João Pedro Rueda Furlan:** Writing – review & editing, Writing – original draft, Formal analysis, Conceptualization. **José Soares Ferreira Neto:** Writing – review & editing. **Marcos Bryan Heinemann:** Writing – review & editing, Formal analysis.

## Funding

The authors thank the São Paulo Research Foundation (grant number 23/16216-4), the National Council for Scientific and Technological Development (grant number 310462/2021), and the Coordination for the Improvement of Higher Education Personnel (grant numbers 88887.463868/2019–00 and Finance Code 001) for fellowships.

## Declaration of competing interest

The authors declare no competing interests.

## Data Availability

The data used to elaborate this paper was obtained from public available PubMed's database.

## Appendix A Supplementary data

Supplementary material Image 1
