# Supplementary material for: The ecological threat posed by invasive species as silent carriers of global priority bacteria to wildlife
Source: One Health. 2025 Apr 18;20:101043. doi: 10.1016/j.onehlt.2025.101043 (PMC12051059; doi:10.1016/j.onehlt.2025.101043)
Supplement: Supplementary file 1 — Supplementary material [file mmc1.docx]

**Supplementary Material**

**The Ecological Threat Posed by Invasive Species as Silent Carriers of Global Priority Bacteria to Wildlife**

**Supplementary Table S1.** Studies investigating WHO priority bacteria and antimicrobial resistance in invasive species

| **Sample collection** | | | **Target** | **WHO priority-related characteristics^1-3^** | | | **Reference** |
| --- | --- | --- | --- | --- | --- | --- | --- |
| **Invasive species** | **Country** | **Year** |  | **Genotype** | **Phenotype** | **ST** |  |
| Giant African snail (*Achatina fulica*) | China | nd | Total DNA | *bla*_CMY,_  *bla*_CTX-M-like_*,*  *bla*_IMP-like,_  *bla*_KPC-like,_  *bla*_NDM-like,_  *bla*_OXA-1-like,_  *bla*_OXA-48-like,_  *bla*_SHV-like,_  *bla*_TEM-like,_  *mecA,*  *mecC,*  *qnrB,*  *qnrS,*  *vanA* | na | na | [1] |
| Green anole  (*Anolis carolinensis*) | Japan | 2009-14 | *Salmonella* spp. | na | nf | na | [2] |
| Marmoset  (*Callithrix sp.*) | Brazil | nd | *Staphylococcus* spp. | nf | nf | na | [3] |
| Urban Pigeon  (*Columba livia*) | Brazil | 2007-08 | *Escherichia coli* | na | nf | na | [4] |
| Urban Pigeon  (*Columba livia*) | South Africa | 2012-13 | *Escherichia coli* | na | 3GC resistance | nd | [5] |
| Urban Pigeon  (*Columba livia*) | Brazil | 2013 | *Escherichia coli*, *Salmonella* spp. | na | nf | na | [6] |
| Urban Pigeon  (*Columba livia*) | Brazil | nd | *Leclercia adecarboxylata* | nf | nf | na | [7] |
| Urban Pigeon  (*Columba livia*) | Brazil | 2018-19 | *Escherichia coli* | *bla*_CTX-M-1_*,*  *bla*_CTX-M-2_*,*  *bla*_CTX-M-8_*,*  *bla*_CMY-2,_  *bla*_TEM-1_ | 3GC resistance | ST10, ST224,  ST457 | [8] |
| Black rat  (*Rattus rattus*) | Brazil | 2018-19 | *Escherichia coli* | *bla*_CTX-M-8,_  *bla*_TEM-1B_ | 3GC resistance | ST10,  ST155 | [8] |
| Urban Pigeon  (*Columba livia*) | South Africa | 2018-22 | *Staphylococcus aureus* | *mecA* | Methicillin resistance | nd | [9] |
| Urban Pigeon  (*Columba livia*) | Bangladesh | 2011 | *Escherichia coli* | *bla*_CTX-M-15_ | 3GC resistance | ST1408,  ST3489,  ST3490,  ST3491 | [10] |
| Urban Pigeon  (*Columba livia*) | Costa Rica | 2013-14 | Total DNA | *bla*_TEM-like_ | nd | nd | [11] |
| Urban Pigeon  (*Columba livia*) | Brazil | 2014-16 | *Escherichia coli* | *bla*_VIM-like_ | Carbapenem resistance | nd | [12] |
| Urban Pigeon  (*Columba livia*) | Australia | 2018 | *Escherichia coli* | *bla*_CTX-M-14,_  *bla*_CTX-M-15,_  *bla*_CTX-M-27_ | 3GC resistance | ST69,  ST93,  ST131,  ST450,  ST695 | [13] |
| Urban Pigeon  (*Columba livia*) | Brazil | 2011-16 | *Escherichia coli* | *bla*_CTX-M-2,_  *bla*_CTX-M-8_ | 3GC resistance | nd | [14] |
| Urban Pigeon  (*Columba livia*) | Brazil | 2012 | *Escherichia coli* | *bla*_CTX-M-8,_  *bla*_TEM-1_ | 3GC resistance | ST359 | [15] |
| Urban Pigeon  (*Columba livia*) | Bangladesh | 2017 | *Salmonella* spp. | na | Fluoroquinolone resistance | nd | [16] |
| Egyptian mongoose (*Herpestes ichneumon*) | Portugal | 2008-11 | *Enterococcus* spp.*,*  *Escherichia coli, Pseudomonas* spp*.,*  *Salmonella* spp*.* | na | nf | na | [17] |
| Asian mongoose (*Herpestes javanicus*) | Japan | nd | *Escherichia coli* | *bla*_TEM-like_ | 3GC resistance | nd | [18] |
| Japanese weasel  (*Mustela itatsi*) | Japan | nd | *Escherichia coli* | *bla*_TEM-like_ | 3GC resistance | nd | [18] |
| American mink  (*Neovison vison*) | Spain | 2018-21 | *Citrobacter* spp., *Escherichia coli*,  *Hafnia alvei,*  *Klebsiella pneumoniae, Morganella morganii,*  *Proteus vulgaris, Providencia stuartii, Salmonella* spp., *Shigella* spp. | *bla*_CMY-2,_  *bla*_CTX-M-15_ | 3GC resistance (*Escherichia coli*) | nd | [19] |
| Pond slider  (*Trachemys scripta*) | Spain | 2018-21 | *Citrobacter* spp., *Enterobacter* spp., *Escherichia coli,*  *Hafnia alvei, Klebsiella* spp., *Morganella morganii*, *Proteus vulgaris, Pseudomonas* spp., *Rahnella aquatilis Salmonella* spp., *Serratia* spp.,  *Shigella* spp. | *bla*_CMY-1,_  *bla*_CMY-2_ | 3GC resistance (*Escherichia* *coli* and *Klebsiella pneumoniae*) | nd | [19] |
| Raccoos  (*Procyon lotor*) | Spain | 2017-19 | *Escherichia coli* | *bla*_SHV-12_ | 3GC resistance | New, but nd | [20] |
| Norway Rat  (*Rattus norvegicus*) | Canada | nd | *Escherichia coli* | na | 3GC resistance | nd | [21] |
| Norway Rat  (*Rattus norvegicus*) and  Black rat  (*Rattus rattus*) | Canada | nd | *Escherichia coli*, *Salmonella* spp. | na | 3GC resistance | nd | [22] |
| Norway Rat  (*Rattus norvegicus*) | Algeria, Argentina, Belgian Congo, Bolivia, Brazil, Burma, Germany, India, Indonesia, Kenya, Kurdistan, Madagascar, Namibia, Senegal, South Africa, Turkey, Uganda, Vietnam, Yemen | 1944-2013 | *Yersinia pestis* | nf | nf | na | [23] |
| Norway Rat  (*Rattus norvegicus*) | United States | 2016-17 | *Enterococcus* spp., *Staphylococcus* spp. | nf | nf | na | [24] |
| Norway Rat  (*Rattus norvegicus*) | Indonesia | 2019 | *Escherichia coli* | *bla*_TEM-like_ | 3GC resistance | nd | [25] |
| Starling  (*Sturnus vulgaris*) | United States | 2007-08 | *Escherichia coli, Salmonella* spp., | na | nf | na | [26] |
| Starling  (*Sturnus vulgaris*) | United States | nd | *Enterococcus* spp., *Staphylococcus* spp. | na | nf | na | [27] |
| Starling  (*Sturnus vulgaris*) | United States | 2012-13 | *Escherichia coli* | *bla*_CMY-2_*,*  *bla*_CTX-M-1_*,*  *bla*_CTX-M-27_*,*  *bla*_CTX-M-32_*,*  *bla*_TEM-1_*,*  *bla*_TEM-141_ | 3GC resistance | ST10,  ST58,  ST69,  ST101,  ST154,  ST155,  ST156,  ST164,  ST224,  ST278,  ST329,  ST345,  ST361,  ST398,  ST539,  ST906,  ST925,  ST1140,  ST1642,  ST2178,  ST2509,  ST2522,  ST4380,  ST4542 | [28] |
| Tramp Ant  (*Tapinoma melanocephalum*) | Brazil | 2005-06 | *Staphylococcus* spp.,  *Klebsiella aerogenes,*  *Hafnia alvei*, *Pseudomonas* sp. | na | nf | na | [29] |

^1^ Antimicrobial resistance investigation as follows: Genotype (conventional and/or quantitative polymerase chain reactions) and phenotype (disk diffusion method). ^2^ Not applied, na; Not found, nf; Third-generation cephalosporin, 3GC. ^3^ The sequence type (ST) was determined by Multilocus sequence typing. Data were retrieved from the PubMed database via National Center for Biotechnology Information interface (accessed on August 16, 2024).

**References**

[1] Y. Zhang, J.-Q. Su, H. Liao, M.F. Breed, H. Yao, H. Shangguan, H.-Z. Li, X. Sun, Y.-G. Zhu, Increasing Antimicrobial Resistance and Potential Human Bacterial Pathogens in an Invasive Land Snail Driven by Urbanization, Environ Sci Technol 57 (2023) 7273–7284. https://doi.org/10.1021/acs.est.3c01233.

[2] D. Sumiyama, A. Shimizu, T. Kanazawa, H. Anzai, K. Murata, Prevalence of *Salmonella* in green anoles (*Anolis carolinensis*), an invasive alien species in naha and Tomigusuku Cities, Okinawa Main Island, Japan, Journal of Veterinary Medical Science 82 (2020). https://doi.org/10.1292/JVMS.19-0594.

[3] I. Sales, O. Vieira-da-Motta, A. Tavares, C.R. Ruiz-Miranda, H. de Lencastre, M. Miragaia, Impact of human created environments in the pathogenic potential and antimicrobial resistance of staphylococci from wild neotropical primates in Brazil, Comp Immunol Microbiol Infect Dis 104 (2024). https://doi.org/10.1016/j.cimid.2023.102094.

[4] V.L. Silva, J.R. Nicoli, T.C. Nascimento, C.G. Diniz, Diarrheagenic *Escherichia coli* strains recovered from urban pigeons (*Columba livia*) in Brazil and their antimicrobial susceptibility patterns, Curr Microbiol 59 (2009). https://doi.org/10.1007/s00284-009-9434-7.

[5] L. Chidamba, L. Korsten, Antibiotic resistance in Escherichia coli isolates from roof-harvested rainwater tanks and urban pigeon faeces as the likely source of contamination, Environ Monit Assess 187 (2015). https://doi.org/10.1007/s10661-015-4636-x.

[6] D. Carvalho, H.C. Kunert-Filho, C. Simoni, L.B. de Moraes, T.Q. Furian, K.A. Borges, J.G. Breunig, L.P. Medeiros, R.K.T. Kobayashi, K.C.T. de Brito, B.G. de Brito, Antimicrobial susceptibility and detection of virulence-associated genes of *Escherichia coli* and *Salmonella* spp. isolated from domestic pigeons (*Columba livia*) in Brazil, Folia Microbiol (Praha) 65 (2020). https://doi.org/10.1007/s12223-020-00781-w.

[7] E. Sano, H. Fontana, F. Esposito, B. Cardoso, B. Fuga, G.C.V. Costa, T.C.M. Bosqueiro, J.A. Sinhorini, L.D. Orico, E. de Masi, C.C. Aires, N. Lincopan, Genomic analysis of fluoroquinolone-resistant *Leclercia adecarboxylata* carrying the ISKpn19-orf-qnrS1-ΔIS3-blaLAP-2 module in a synanthropic pigeon, Brazil, J Glob Antimicrob Resist 33 (2023) 256–259. https://doi.org/10.1016/j.jgar.2023.04.013.

[8] E. Sano, F. Esposito, H. Fontana, B. Fuga, A. Cardenas-Arias, Q. Moura, B. Cardoso, G.C.V. Costa, T.C.M. Bosqueiro, J.A. Sinhorini, E. de Masi, C.C. Aires, N. Lincopan, One health clones of multidrug-resistant *Escherichia coli* carried by synanthropic animals in Brazil, One Health 16 (2023) 100476. https://doi.org/10.1016/j.onehlt.2022.100476.

[9] T.K. Wilson, O.T. Zishiri, M.E. El Zowalaty, Molecular detection of multidrug and methicillin resistance in *Staphylococcus aureus* isolated from wild pigeons (*Columba livia*) in South Africa, One Health 18 (2024). https://doi.org/10.1016/j.onehlt.2023.100671.

[10] B. Hasan, K. Islam, M. Ahsan, Z. Hossain, M. Rashid, B. Talukder, K.U. Ahmed, B. Olsen, M. Abul Kashem, Fecal carriage of multi-drug resistant and extended spectrum β-lactamases producing *E. coli* in household pigeons, Bangladesh, Vet Microbiol 168 (2014). https://doi.org/10.1016/j.vetmic.2013.09.033.

[11] K. Blanco-Peña, F. Esperón, A.M. Torres-Mejía, A. de la Torre, E. de la Cruz, M. Jiménez-Soto, Antimicrobial Resistance Genes in Pigeons from Public Parks in Costa Rica, Zoonoses Public Health 64 (2017). https://doi.org/10.1111/zph.12340.

[12] T.S. Bueno, M.R. Loiko, M.R. Vidaletti, J.A. de Oliveira, T. Fetzner, C. Cerva, L.B. de Moraes, S. De Carli, F.M. Siqueira, R.O. Rodrigues, M. de M. Coppola, S.M. Callegari-Jacques, F.Q. Mayer, Multidrug-resistant *Escherichia coli* from free-living pigeons (*Columba livia*): Insights into antibiotic environmental contamination and detection of resistance genes, Zoonoses Public Health 69 (2022). https://doi.org/10.1111/zph.12957.

[13] S. Mukerji, S. Gunasekera, J.N. Dunlop, M. Stegger, D. Jordan, T. Laird, R.J. Abraham, M. Barton, M. O’Dea, S. Abraham, Implications of Foraging and Interspecies Interactions of Birds for Carriage of *Escherichia coli* Strains Resistant to Critically Important Antimicrobials, Appl Environ Microbiol 86 (2020). https://doi.org/10.1128/AEM.01610-20.

[14] M.P.V. Cunha, M.C.V. Oliveira, M.G.X. Oliveira, M.C. Menão, T. Knöbl, CTX-M-producing *Escherichia coli* Isolated from urban pigeons (*Columba livia domestica*) in Brazil, J Infect Dev Ctries 13 (2019). https://doi.org/10.3855/JIDC.11441.

[15] C.A. Borges, R.P. Maluta, L.G. Beraldo, M. V. Cardozo, E.A.L. Guastalli, S. Kariyawasam, C. DebRoy, F.A. Ávila, Captive and free-living urban pigeons (*Columba livia*) from Brazil as carriers of multidrug-resistant pathogenic *Escherichia coli*, Veterinary Journal 219 (2017). https://doi.org/10.1016/j.tvjl.2016.12.015.

[16] S.J.I. Karim, M. Islam, T. Sikder, R. Rubaya, J. Halder, J. Alam, Multidrug-resistant *Escherichia coli* and *Salmonella* spp. Isolated from pigeons, Vet World 13 (2020). https://doi.org/10.14202/vetworld.2020.2156-2165.

[17] M.V. Cunha, T. Albuquerque, P. Themudo, C. Fonseca, V. Bandeira, L.M. Rosalino, The gut microbiota of the Egyptian mongoose as an early warning indicator of ecosystem health in Portugal, Int J Environ Res Public Health 17 (2020). https://doi.org/10.3390/ijerph17093104.

[18] I. Nakamura, T. Obi, Y. Sakemi, A. Nakayama, K. Miyazaki, G. Ogura, M. Tamaki, T. Oka, K. Takase, A. Miyamoto, Y. Kawamoto, The prevalence of antimicrobial-resistant *Escherichia coli* in two species of invasive alien mammals in Japan, Journal of Veterinary Medical Science 73 (2011). https://doi.org/10.1292/jvms.10-0525.

[19] T.S. Mengistu, B. Garcias, G. Castellanos, C. Seminati, R.A. Molina-López, L. Darwich, Occurrence of multidrug resistant Gram-negative bacteria and resistance genes in semi-aquatic wildlife - *Trachemys scripta*, *Neovison vison* and *Lutra lutra* - as sentinels of environmental health, Science of the Total Environment 830 (2022). https://doi.org/10.1016/j.scitotenv.2022.154814.

[20] J.A. Orden, I. García-Meniño, S.C. Flament-Simon, J. Blanco, R. de la Fuente, A. Martínez-Rodrigo, A. Mas, J. Carrión, F. Sobrino, G. Domínguez-Bernal, Raccoons (*Procyon lotor*) in the Madrid region of Spain are carriers of antimicrobial-resistant *Escherichia coli* and enteropathogenic E. coli, Zoonoses Public Health 68 (2021). https://doi.org/10.1111/zph.12784.

[21] C.G. Himsworth, E. Zabek, A. Desruisseau, E. Jane Parmley, R. Reid-Smith, M. Leslie, N. Ambrose, D.M. Patrick, W. Cox, Avian pathogenicity genes and antibiotic resistance in *Escherichia coli* isolates from wild norway rats (*Rattus norvegicus*) in British Columbia, Canada, J Wildl Dis 52 (2016). https://doi.org/10.7589/2015-09-238.

[22] C.G. Himsworth, E. Zabek, A. Desruisseau, E. Jane Parmley, R. Reid-Smith, C.M. Jardine, P. Tang, D.M. Patrick, Prevalence and characteristics of *Escherichia coli* and *Salmonella* spp. In the feces of wild Urban Norway and black rats (*Rattus norvegicus* and *Rattus rattus*) from an inner-city neighborhood of Vancouver, Canada, J Wildl Dis 51 (2015). https://doi.org/10.7589/2014-09-242.

[23] N. Cabanel, C. Bouchier, M. Rajerison, E. Carniel, Plasmid-mediated doxycycline resistance in a *Yersinia pestis* strain isolated from a rat, Int J Antimicrob Agents 51 (2018). https://doi.org/10.1016/j.ijantimicag.2017.09.015.

[24] G.R. Gerbig, H. Piontkivska, T.C. Smith, R. White, J. Mukherjee, H. Benson, M. Rosenbaum, J.H. Leibler, Genetic characterization of *Staphylococcus aureus* isolated from Norway rats in Boston, Massachusetts, Vet Med Sci 9 (2023). https://doi.org/10.1002/vms3.1020.

[25] H. Le Huy, N. Koizumi, H. Nuradji, Susanti, S.M. Noor, N.L.P.I. Dharmayanti, T. Haga, K. Hirayama, K. Miura, Antimicrobial resistance in *Escherichia coli* isolated from brown rats and house shrews in markets, bogor, Indonesia, Journal of Veterinary Medical Science 83 (2021). https://doi.org/10.1292/jvms.20-0558.

[26] S.M. Gaukler, G.M. Linz, J.S. Sherwood, N.W. Dyer, W.J. Bleier, Y.M. Wannemuehler, L.K. Nolan, C.M. Logue, *Escherichia coli*, *Salmonella*, and *Mycobacterium avium* subsp. *paratuberculosis* in Wild European starlings at a Kansas cattle feedlot, Avian Dis 53 (2009). https://doi.org/10.1637/8920-050809-Reg.1.

[27] J. Anders, B. Bisha, High-Throughput detection and characterization of antimicrobial resistant *Enterococcus* sp. isolates from GI tracts of european starlings visiting concentrated animal feeding operations, Foods 9 (2020). https://doi.org/10.3390/foods9070890.

[28] J.C. Chandler, J.E. Anders, N.A. Blouin, J.C. Carlson, J.T. LeJeune, L.D. Goodridge, B. Wang, L.A. Day, A.M. Mangan, D.A. Reid, S.M. Coleman, M.W. Hopken, B. Bisha, Author Correction: The Role of European Starlings (*Sturnus vulgaris*) in the Dissemination of Multidrug-Resistant *Escherichia coli* among Concentrated Animal Feeding Operations (Scientific Reports, (2020), 10, 1, (8093), 10.1038/s41598-020-64544-w), Sci Rep 10 (2020). https://doi.org/10.1038/s41598-020-68365-9.

[29] M.M. Teixeira, A. Pelli, V.M. dos Santos, M. das G. Reis, Microbiota associated with tramp ants in a Brazilian University Hospital, Neotrop Entomol 38 (2009). https://doi.org/10.1590/s1519-566x2009000400017.
